# The influence of indigenous knowledge on chemistry metacognition

**DOI:** 10.12688/f1000research.131685.4

**Published:** 2024-02-29

**Authors:** Tavonga Tawanda, Awelani V. Mudau

**Affiliations:** 1 science and technology education, university of south africa, pretoria, gauteng, South Africa

**Keywords:** academic performance, African philosophical orientation, chemistry metacognition, contextualisation, indigenous chemistry knowledge.

## Abstract

**Background:**

Chemistry is viewed as a difficult and challenging subject by many learners and teachers which leads to poor academic performance in the subject. The majority of the pre-service science teachers in Zimbabwean teachers’ colleges also find Chemistry to be a challenging subject. The focus of this study was to simplify and contextualize the teaching and learning of Chemistry concepts for life-long survival and problem-solving skills through exploring the influence of indigenous Chemistry knowledge on Chemistry metacognition.

**Methods:**

An embedded mixed methods case study was underpinned by the social constructivist theory, which is used to collect and analyse the data. Twenty-nine respondents were purposively sampled. Their metacognition awareness was determined through focus group interviews which are triangulated with a paper and pen test. The indigenous Chemistry knowledge possessed by the pre-service science teachers was collected using focus group interviews, which was then used in the intervention stage for Chemistry metacognition.

**Results:**

The findings suggest that indigenous knowledge influences chemistry metacognition in a positive way.

**Conclusions:**

Further research is required on the relationship between indigenous Chemistry knowledge and Chemistry metacognition. It is recommended that Chemistry educators should be capacitated with skills for identifying and applying indigenous Chemistry knowledge that is relevant to Chemistry metacognition.

## Introduction

The learning in the schools and other educational institutions are influenced by the cultural background of leaners which inturn infleunces their learning style ( Smith, Kayser, Wallace, Bosnake, 2023; Baker and Taylor, 1995). The complex and diverse local, technical indigenous, traditional, peasant, environmental, folk knowledge including empirical knowledge which were acquired through practice, observation and transmitted orally from generation to generation to meet the needs of a particular indigenous society by a dynamic process are called indigenous knowledge systems ( da Silva, Pereira and Amorim, 2023). Indigenous Knowledge System (IKS) refers to complicated knowledge systems attained and passed from generations to generations by local society as through interaction with their local environment encompasing philosophical,technological, economic, governance and learning systems ( Chikaire, Osuagwu, Ihenacho, Oguegbuchulam, Ejiogu-Okereke, and Obi, 2012). IKS consists of beliefs and a body of knowledge that is empirical which was passed down through generations by a particular community of a specific local environment using cultural transmission with an interrelation amongst the living things and with their environment ( Chikaire, Osuagwu, Ihenacho, Oguegbuchulam, Ejiogu-Okereke, and Obi, 2012).

A very important point is to emphasize the incompleteness that is present in all knowledge systems and the huge potential for creating discourse between empirical indigenous knowledge systems and western science with their integration leading to a more culturally sensitive and holistic approach to science teaching and learning boosting learners’ understanding of western science. This would be a practical way of school science curriculum indigenization which increases student success through cognitive development leading to great academic performance ( Govender and Mutendera, 2020).

There are some common characteristics between indigenous chemistry and western chemistry knowledge such as being rational, ideological, communal and empirical ( Aikenhead and Jegede, 1999). These similarities were confirmed by who saw indigenous chemistry knowledge and western chemistry knowledge both having characteristics such as; being able to give explanations of complex systems, basing on observations, verifying through repetition, seeking the understanding of the of physical world and changing of the body of knowledge over time ( Baker, Rayner, and Wolowic, 2011). This allows indigenous chemistry knowledge and western chemistry knowledge to complement each other in terms of chemistry knowledge and skills construction and transmission.

This results in two Chemistry knowledge perspectives (worldviews), indigenous cultural Chemistry knowledge and western cultural Chemistry knowledge which differ only on cultural perspective/views. Chemistry is a sub-culture of Western culture ( Cobern and Aikenhead, 1997). African science learners in Sub-Sahara Africa experience many challenges in science education associated with the clashes between western science which is Eurocentric, and African indigenous knowledge due to the two knowledge systems’ social and cultural differences ( Mhakure and Mushaikwa, 2014). Emphasis must be on the importance and benefits of identifying similarities between indigenous Chemistry and western Chemistry ways of knowledge construction and transmission to create an understanding of the natural world, which is more encompassing ( Tsuji and Ho, 2002). It is crucial to have a blending of indigenous Chemistry knowledge and western Chemistry knowledge learning, which leads to an environment of shared learning whereby indigenous Chemistry knowledge is not assimilated by the dominant western Chemistry knowledge, but is thoroughly articulated and clearly heard, resulting in learning being reciprocal and making Chemistry learning meaningful to local Chemistry learners ( Banner, 2016).

Philosophically, indigenous Chemistry knowledge is a form of knowledge that is generated locally which is real and disseminated through learning from social and environmental interactions by pre-service science teachers. Social constructivists view cognitive functions as dependent on social interactions with other individuals and the environment ( Masciotra, 2005). Indigenous knowledge is a form of reality which comes from social invention that has solved and continues to solve Chemistry challenges in indigenous society. Social interactions and cultural influences result in shared meaning of indigenous Chemistry knowledge making it a human product, as it has been tested over time and there is physical as well as chemical evidence of it and its uses ( Achimugu, Fasanya, Abdulwaheed, Joshua, Ibrahim, and Shaibu, 2023; Mudau and Tawanda, 2022; Zidny and Eilks, 2020). Learning is a social process which in itself is active knowing that involves the activation and application of prior knowledge. Prior knowledge is the basis for all learning. Indigenous Chemistry knowledge is the prior knowledge which is a basis of learning. The advanced deliberate manipulation of mental or emotional effects of perception and reasoning directed towards a particular goal such as improving learning, which is the acquisition of knowledge, skills and affective states is called metacognition ( Teng, 2023; Sümeyye and Egmir, 2022). Metacognition is recognition of the value of prior knowledge whilst chemistry metacognition is the recognition of the value of indigenous Chemistry knowledge (prior-knowledge) ( Taylor, 1999).

**Figure 1.**
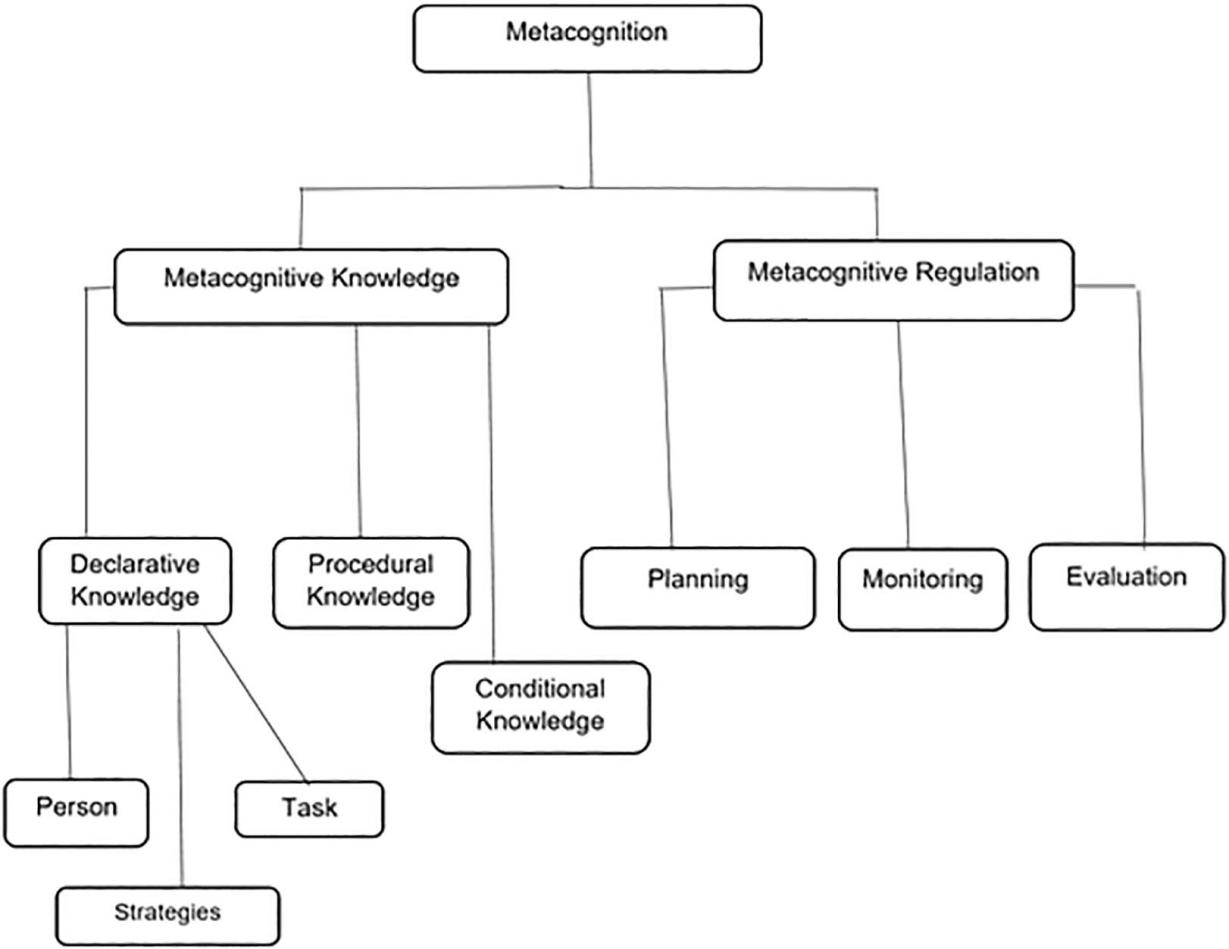
Metacognition and its components.

There are two major components of metacognition which are further divided into other subcomponents. Metacognitive knowledge (cognitive knowledge) and metacognitive regulation (cognitive regulation) make up metacognition ( Nazarieh, 2016; Lai, 2011; Noushad, 2008). Three aspects of knowledge comprise metacognition, that is, procedural knowledge and conditional knowledge which are closely related as well as declarative knowledge, which refers to knowing in terms of knowledge, strategies and skills which is important for completing a learning task successfully under different conditions ( Mahdavi, 2014; Schraw, 2000). This is knowledge about the task at hand in terms of prior knowledge, which is useful in the given scenario. Declarative knowledge is divided into person, task and strategies (actions) variables ( Flavell, 1979). Person variables involve recognition of one’s strengths and weaknesses in the learning process, including information processing. Task variables refer to what an individual knows or might find out in terms of the nature and mental (intellectual) requirements (demands) to accomplish the learning task points out ( Papaleontiou-Louca, 2019). Metacognitive control/regulation of cognition or executive control are sequences of activities that assist learners to control their own learning or thinking ( Nazarieh, 2016). Metacognitive control as having three components or skills which are planning, monitoring and evaluation ( Schraw and Dennison, 1994). Planning includes the choosing of befitting strategies and provisions that are effective in terms of performance or goal attainment. Monitoring is the judgement of the progress of one’s current thinking and task performance. Evaluation refers to assessing or examining the completed task or goal which can demand more planning, monitoring and evaluating depending on the outcome. Metacognition and its components are shown in Figure 1.

Metacognition has a positive impact in general on learner outcomes in terms of thinking and learning, particularly for learners with disabilities ( Georghiades, 2004). Metacognition promotes independent Chemistry knowledge, knowledge that is more permanent, motivates learners and improves educational achievement across different ages, intellectual abilities and subject areas ( Smith, Black and Hooper, 2017; Somerville, 2017; Philling-Cormick and Garrison, 2007; Louca, 2003). Learners with high academic achievement have more metacognitive awareness and partake in a lot of self-regulation compared to lower academic achieving learners ( Hartman, 2001). All learners are capable of metacognition as long as they have the ability to perform a skill ( Schraw, 1998). In learners, metacognition is promoted through awareness by learners of the existence of metacognition, which differs from cognition, and thus academic success is increased ( Schraw, 1998). Cognition are mental processes that are invloved in understanding, knowing, decision making, learning, attention, problem-solving and memory whilst metacognition is the knowledge and active control of mental processes that are invloved in understanding, knowing, decision making, learning, attention, problem-solving and memory ( Stanton, Sebesta and Dunlosky, 2021; Papaleontiou-Louca, 2008). An interactive approach that mixes teacher and expert modelling, direct instruction, group activities which allow learners to share the knowledge they have on cognition and reflection can be extremely effective ( Schraw, 1998).

The perception held by many learners and teachers is that Chemistry is a difficult and challenging subject ( Uzezi, Ezekiel and Auwal, 2017; Mahdi, 2014; Gafoor and Shilna, 2013). In Matabeleland North and other Zimbabwean provinces, the biology, Chemistry and physics subjects are believed to be extremely difficult by both learners and teachers ( Regional Centre for Social Responsibility, 2012; Ogunkola and Samuel, 2011). A study in Zimbabwe showed that science education suffers because of a negative attitude from learners ( Gadzirayi, Bongo, Ruyimbe, Bhukuvhani and Mucheri, 2016). The perception of Chemistry being exceedingly difficult is even stronger in rural secondary day-schools. Rural secondary schools are government-owned schools in rural areas, which are normally poorly resourced in terms of both human and material resources that negatively affects the teaching and learning process. Due to this perception, Chemistry is viewed as a subject for very intelligent or gifted learners only ( Cardellini, 2017; Fu, Fitzpatrick, Conners, Clay, Toombs, Busby and Discoll, 2015). As a result, very few learners take up Chemistry as a subject in their studies. For the few who take it up, their performance is usually poor when compared to other subjects ( Thondhlana, 2020). There are concerns in Tsholotsho North in Matabeleland North about the poor performance by learners in pure sciences and mathematics at ordinary level in Zimbabwe ( Dube, 2016). There is poor academic performance in sciences, especially in Chemistry in general ( Uchegbu, Oguoma, Elenwoke and Ogbuagu, 2016; Hassan, Ali, Salum, Kassim, Elmoge and Amour, 2015; Anditi, Okere and Muchiri, 2013; Ezeudu and Obi, 2013).

### Research problem

Pre-service science teachers find Chemistry difficult and challenging as they struggle with the Chemistry knowledge, skills and attitudes, thus resulting in poor academic performance in Chemistry at teacher education institutions ( Adu-Gyamfi and Asaki, 2023; Bicak, Borchert and Höner, 2021; Mudzakir, 2017; Wheeldon, 2017; Yalcin-Celik,Kadayifci, Uner, and Turan-Oluk, 2017; Cam, Topcu and Sulun, 2015). The researchers’ observation during the August Vacation School teaching was that there was indeed poor performance by learners in Chemistry. The majority of the pre-service science teachers at the teachers’ college come from Matabeleland province schools, which have low pass rates and have learners who find Chemistry challenging ( Chikiwa, 2018; Nkala, 2018). Pre-service science teachers find Chemistry difficult and challenging as they struggle with the Chemistry knowledge, skills and attitudes, thus resulting in poor academic performance in Chemistry at the teachers’ college ( Mudzakir, 2017; Wheeldon, 2017; Cam, Topcu and Sulun 2015). Misconceptions or alternative conceptions, insufficient content knowledge, skills and attitudes contribute significantly to the pre-service science teachers’ ability to adequately learn Chemistry in terms of difficulties and challenges. Pre-service teachers were found to have electrochemistry misconceptions and inadequate content knowledge ( Yilmaz and Bayrakceken, 2015; Bektas, Tuysuz, Kirbulut and Cetin-Dindar, 2011). The teaching and learning of Chemistry concepts are detached from the socio-economic daily life experiences of pre-service science teachers ( Ugwu and Diovu, 2016) as it is conceptualised from the western and eastern philosophical orientations that are based on a universal approach towards knowledge generation and dissemination ( Shumba, 2014).

### Research focus

Internationally and regionally, research has shown that the acquisition and use of metacognition improves academic achievement and motivation of learners including, those who are intellectually limited, education resource disadvantaged, prior knowledge limited as well as culturally disadvantaged. A study of how the performance of secondary school students in Chemistry was impacted by metacognition awareness was studied in the Asian country of Pakistan ( Rahman, Jumani, Chaudry, Chitsti and Abbasi, 2010). The findings were that; (i) metacognition is important in student’s academic achievement and, (ii) highly metacognitive students’ performance was better than those of low metacognitive skills ( Rahman, Jumani, Chaudry, Chitsti and Abbasi, 2010). The function of metacognition among primary school learners in the African country of Kenya in everyday problem solving was studied ( Aurah, Koloi-Keaikitse, Isaacs and Finch, 2011). The findings of the study were; (i) learners who were better problem solvers were those with higher metacognition level than learners who had low levels of metacognition and, (ii) high grade level learners were better in terms of metacognition and the ability to perform problem-solving ( Aurah, Koloi-Keaikitse, Isaacs and Finch, 2011). Van Aswegen (2015) did a study on the metacognitive awareness development of intermediate phase young learners using stories for facilitation in South Africa. The finding of the research implied that the development of metacognitive awareness using story-based interventions yielded positive results ( Van Aswegen, 2015).

Most of the research that has been done has focused on the relationship between metacognition and academic achievement. However, there is a gap in knowledge on the contribution of indigenous Chemistry knowledge (pre-service science teachers’ prior knowledge and beliefs) to Chemistry metacognition in the Zimbabwean teachers’ college context. This research seeks to fill that gap in knowledge. The ideal Chemistry teaching and learning should be done from the African philosophical orientation that is locally contextualised and culturally relevant in terms of knowledge generation and dissemination that meets the needs and provides practical solutions to the everyday life chemical challenges and problems of the community ( Oladejo, Okebukola, Olateju, Akinola, Ebisinand Dansu, 2022; Verharen, Gutema, Tharakan, Bugarin, Fortunak, Kadoda, Liu, and Middendorf, 2014; Buchanan, 2012).

### Research aim and research questions

The aim of this research was to explore the use of culturally contextualised indigenous Chemistry knowledge by pre-service science teachers in Chemistry metacognition. The following research questions guided the study:

(i) What is the indigenous Chemistry knowledge possessed by pre-service science teachers?
(ii) How relevant is the indigenous Chemistry knowledge to Chemistry metacognition?
(iii) How effective is the indigenous Chemistry knowledge in Chemistry metacognition?

## Methods

### General background

An embedded mixed methods case design was employed which had 99% of qualitative and 1% of quantitative elements in which the interpretive paradigm was employed to study the influence of indigenous Chemistry knowledge on Chemistry metacognition. Focus group interviews and a test (paper and pen) were used to assess the metacognitive awareness level of the pre-service science teachers before and after the indigenous Chemistry knowledge intervention. Focus group interviews were also used to get the indigenous Chemistry knowledge possessed and practised by pre-service science teachers. This study was conducted with post ordinary level pre-service science teachers from a secondary teacher training college in Zimbabwe.

### Ethical considerations

The University of South Africa college of education ethics review committee approved the study on 2019/07/24, and consent was sort of in a written form. The certificate has the following reference number 2019/07/24/63816997/18/MC. This article is written in in with SRQR guidelines ( Mudau, 2023d).

### Sample

The participants were 29 (15 males and 14 females) post ordinary level pre-service science teachers who were in their first term at college who had no previous exposure to tertiary chemistry education. The whole class was asked to participate and all agreed. There was no exclusion criteria. The highest academic qualification for 20 of the pre-service science teachers was ordinary level and 9 was advanced level. There are three major levels of education in Zimbabwe which are primary, secondary and tertiary level. The primary level consists of 9 years of schooling that are split into 4 years of infant education and 5 years of junior education which has an external public examination written at the end. Secondary level is split into junior high school and senior high school. Junior high school is 4 years which ends with an external public examination called ordinary level examination. A full certificate at ordinary level must have five subjects passed including English language, Mathematics and Combined Science (formally Integrated Science). Combined Science consists of Biology, Chemistry and Physics at General Certificate of Secondary Education level.

The ordinary level full certificate enables learners to: look for work, proceed to senior high school or go to tertiary institutions for professional training such as teachers’ colleges. Senior high school (advanced level) is two years (form 5 and 6) and is done after having passed five ordinary level subjects. Advanced level external public examinations are written in the second year (form 6) of senior high school. A complete certificate at advanced level consists of two subjects passed which enables learners to look for work or enroll at tertiary institutions.

Purposive sampling was used to choose the participants. All the students in a particular class in the college of education were asked to participate using letters. They all accepted. Out of the 29 participants, those who had chemistry at ordinary level were five and those with chemistry at advanced level were 3 who were also part of the 5 who had chemistry at ordinary level.

### Instrument and procedures

The first author who is male and held a master degree at the time was the lecturer at the institution from which data was collected had to collected all the data. The data was part of his PhD studies. The first author gave the potential participants consent letters that were approved by the ethical committee of the university. The participant consent letters contained the purpose of the research and why they were requested to participate. Pre-service science teachers were chosen in a manner that was representative of the pre-service science teachers population and were randomly assigned to focus groups. Specific procedures were adhered for each pre-service science teacher and each focus group. Methodology triangulation was used to ensure that matters of biases are taken care of. The second author also read the transcripts to ensure that there was internal validity. In stage one, the metacognitive awareness levels of the pre-service science, teachers before the indigenous chemistry knowledge intervention were ascertained using an adapted metacognitive awareness inventory from Schraw and Dennison (1994) . The adaption was from a metacogitive awareness inventory to a chemistry metacognition awareness inventory. The adapted metacognitive awareness inventory was in the form of focus group interview guide and paper and pen test for triangulation purposes. So the students were given the pen and pare test first and thereafter they were also interviewed. As indicated what was in the pen and paper instrument was also similar to what was in the interview guide. The tools were piloted to ensure that they were free to a certain degree of any biases or mistakes. This happened after contact teaching time. This happened between March and June in 2020. The adapted Metacognitive Awareness Inventory (MAI) ascertained the levels of all the metacognition sub-components which were: declarative knowledge, procedural knowledge, conditional knowledge (knowledge of cognition) and planning, information management strategies, comprehension monitoring, debugging strategies and evaluation (regulation of cognition).

In stage two, focus group interviews conducted by the first author were used for identifying the cultural indigenous Chemistry knowledge held and practised by pre-service science teachers. Each focus group had a maximum of nine participants and they were three groups with two of those having 10 students each. The interviews were recorded by an audio recording gadget. Each group varied from between 20 minutes to 30 minutes in duration. For all the tools that were used for this study field notes were also taken for triangulation purposes. Document analysis was also used for identifying the cultural indigenous Chemistry knowledge held and practised by pre-service science teachers in stage two. In stage three, focus group interviews and paper and pen test were used to assess the extent to which pre-service science teachers have acquired Chemistry metacognition after the cultural indigenous Chemistry knowledge intervention. The intervention was a series of four additional lesson wherein based on what was discovered from the stage 1 and 2 data collection procedures. The content ranged from fermentation processes from their homes to fractional distillation of liquid air process from their homes.

Observations were done with pre-service science teachers in the Chemistry laboratory at the teachers’ college during Chemistry lecture times. Permission was asked for using the letters and consent forms as approved by the ethics committee. Document analysis was done using pre-service science teachers’ written tests, assignments and practical write-ups at the teachers’ college after marking the pieces of work. For stage four, document analysis was used to assess and find out the extent to which pre-service science teachers have acquired Chemistry metacognition through cultural indigenous Chemistry knowledge. The pieces of work that focused on the topics of atomic structure, redox reactions, stoichiometry, periodic table and periodicity as well as practical assessments were analysed. This was done over a period of eight weeks in which three tests, two assignments and two practical write-ups were done. The college minimum assessment requirements are one test, 1 assignment and 1 practical per term.

Materials used in this study can be found as *Extended data* ( Mudau, 2023c).

### Data analysis

The typology approach which was a constant comparison data analysis was used to analyse the metacognitive awareness levels of the pre-service science teachers before and after the indigenous chemistry knowledge intervention. In a typology approach themes and categories are predetermined but this does not mean that if new themes and categories are identified whilst analysing data they are not included. The data analysis scheme was developed based on the research questions and literature reviewed. The indigenous chemistry knowledge possessed and practised by the pre-service science teachers for use in the indigenous chemistry knowledge intervention was also analysed using the constant comparison data analysis. Quantitative data was analysed using SPSS version 23. The metacognition awaresness inventory has 52 questions which require true/false responses. The scoring is done in such a way that the “true” response earns 1 mark and the “false” response earns 0 marks. These metacognition awareness questions are grouped into metacognition subcomponents: declarative knowledge (possible score 8), procedural knowledge (possible score 4), conditional knowledge (possible score 5), planning (possible score 7), comprehension monitoring (possible score 7) information management strategies(possible score 10, debugging strategies (possible score 5) and evaluation strategies (possible score 6). These qualitative questions results in quantitative answers which are then processed using the Statistical Package for Social Sciences (SPSS) Version 3, using SPSS’S statistical program for quantitative data analysis comparison tool to convert this data into line graphs, bar graphs and pie charts for analysis. The chemistry metacognition awareness data before and after intervention is compared using the line graphs, bargraphs and pie charts.

## Results

The results are presented below from the sample described in the methodology. The raw results can be found as *Underlying data* ( Mudau, 2023a, b).

### What is the indigenous Chemistry knowledge possessed by pre-service science teachers?

Pre-service science teachers exhibited that they are a repository for indigenous Chemistry knowledge and skills. Indigenous Chemistry knowledge and skills possessed by pre-service science teachers cover most survival areas in their lives. Extensive use and effectiveness of indigenous Chemistry knowledge and skills were found in agriculture, environmental conservation, food processing, food preservation and health care. These verisimilitudes indigenous knowledge, skills and attitudes are shown in Tables 1 to 6.

**Table 1.** Agriculture indigenous knowledge and skills.

**Agriculture indigenous knowledge and skills practised by pre-service science teachers’ communities of origin**
*Use of manure as a fertiliser. Use of ashes as a pest control such as maize stalk borer. Ploughing using cattle. Rearing animals (cattle, sheep, goats, pigs). Crop rotation, paddocking. Nhimbe -other villagers assist in ploughing, planting, cultivation and harvesting. Cultivation using hoes. Mixed farm planting-a variety of crops in the same field. Milking of cattle. Hand picking of locusts. Ox drawn ploughs. Crops, chicken and cattle rearing. Removing weeds using cultivation. Land preparation - cutting down trees. Hunting using catapults and dogs. Using cattle for ploughing. Use ashes to remove aphids from vegetables.* *Use of chickens to get rid of locusts (predator method) without harming the plants. Use of ashes as fertiliser. Weeding and ploughing. Growing sorghum and millet. Planting seeds from the previous produce. Organic agriculture. Use aloe vera to control pests on livestock. Subsistence farming. Keeping road runners for commercial use. Fishing using hooks and nets. Farming vegetables, harvesting, hunting wild animals for meat, gathering fruits and edible roots. Planting sorghum or millet. Using plant litter to make compost (recycling). Mulching. Cross breeding. How to plant sweet potatoes. Herding cattle. Fishing using nets, traps, fishing rods and herbs used to kill fish.*

**Table 2.** Indigenous environmental conservation knowledge and skills.

**Indigenous environmental conservation knowledge and skills practised by pre-service science teachers’ communities of origin**
*Creating ridges to prevent soil erosion. Planting trees to avoid de-forestation. Crop rotation as different plants use different minerals. Cow dung used as floor polish. Ploughing before rain season to preserve nutrients. Headman prevents villagers from cutting down trees. Tree planting-prevents soil erosion. Fire guards. Plant trees (indigenous). Do not cut trees that are in your homestead (Mukusu) or immediately after the homestead. Afforestation- growing trees were they never existed before. Not eating an animal with the name similar to your totem (For example Mpofu would not eat an eland). Application of rocks on gullies, contour ridges to prevent soil erosion. Use of sticks to prevent gully formation. Use of contour ridges and filling up gullies with sticks.*

**Table 3.** Indigenous food processing knowledge and skills.

**Indigenous food processing knowledge and skills practised by pre-service science teachers’ communities of origin**
*Boiling and drying, salting. Boiling of milk. Drying vegetables/meat and drying sweet reeds. Grinding grain using a mortar and pestle. Pounding grain to make mealie meal. Drying mealies. Smoking (meat), Caterpillar (mopani worms) preparation - remove insides, boil, salt and dry. Meat salting. Dehydration of food. Sweet potatoes - pfimbi - kept in a hole and covered with ashes. Use of ashes (soda) to cook okra. Fermenting marula fruit. Maize cooked, dried and put in kitchen so that it is smoked. Making (grinding) peanut butter using a mortar and pestle. Production of peanut butter using stones (mortar and pestle). Brewing of beer and wine.* *Making soap. Drying vegetables (ulude). Making biltong. Processing (fermenting) milk into sour milk. Cooking (Idelele) okra. Preparing amangqina (cow hooves). Preparing porridge (nhopi) from pumpkin/melon. Dried meat (biltong). Milking and keeping the milk fresh. Umxhanxa (food)- mixture of melon and dried mealies. Umcaba - amasi mixed with amabele. Salting food (meat, fish). Brewing. Mahewu (indigenous non- alcoholic drink). Winnowing. Brewing tototo, thothotho (an illicit alcoholic brew through fermentation and distilling). Chimodo (homemade bread from wheat and maize-meal). Cooking sadza (thick maize meal). Wine preparation (tototo, marula, opaque beer). Chimodo - home bread - wheat, mealie - meal.*

**Table 4.** Indigenous food preservation knowledge and skills.

**Indigenous food preservation knowledge and skills practised by pre-service science teachers’ communities of origin**
*Place meat in a bowl/tin and place in a sack that is moist. Smoking. Boiling and salting. Boiling. Clay pot placed in sand and water poured to preserve food. Fermenting marula fruit to make wine and fresh milk to sour milk. Maize cooked, dried and put in kitchen so that it is smoked. Smoking meat, drying vegetables and meat. Placing food in a hole in the ground to keep it fresh and cover with soil. Drying and smoking. Sweet reeds are dried; they are sweeter than wet/fresh ones. Building traditional silos. Drying grain, store in silos. Vegetable – preservation - cut it up and dry. Preservation of food by drying (mfushwa - dried vegetables).*

**Table 5.** Indigenous health care knowledge and skills.

**Indigenous health care knowledge and skills practised by pre-service science’ communities of origin**
*When a cow udder is not releasing milk for the calf, herbs and a chicken feather are used to make the cow udder release milk as the chicken feather is inserted into the cow udder overnight after applying the herbs. Intolwane - Herb for treating excess acid and wind in the stomach. Traditional healers-herbs, isihaqa treats stomach acids. Isihaqa - herb used for treating excess bile. Marula bark-strengthening bone. Guchu-mixture of herbs (msasa, murumanyama) treats sextual infections. Marula bark - used by women who recently gave birth to treat loose vagina so as to tighten the vagina. Use of black jack to treat stomach pains.* *For a cough - boil thetshane and drink. Stomach problems – drink aloe vera. Use a hot iron rod on the eye of a cow for a sight problem. Drink unshashanyama/isihaqa for stomach pains or running stomach. Marula/Nyama bark/charcoal- used to treat running stomach. Using honey to prevent asthma attacks. Use of ashes to heal wounds. Use of marula bark to relieve constipation. Treat infertility in humans using mupapama. Herbs – isihlalutho- used to treat tooth ache, isihaqa-treating stomach ache, enhance sexual performance, intolwane - treats stomach as well as excess air or gas in the stomach. Bark of marula tree cures stomach acids. Use of natural herbs-coughs, stomach acids, headaches. Herbs for nose bleeding. Grinding using a mortar and pestle. Survival skills – when you get injured you know which herbs to go for.*

**Table 6.** Other indigenous chemistry knowledge and skills.

**Indigenous Chemistry knowledge**
*Brick moulding. Baking. Water harvesting. Sawing. Hunting. Soso - a plant used for shampooing hair. How to preserve the body of the deceased? Rekindle fire through blowing using the mouth. Salt - brushing the teeth. Cow dung – as floor polish. Basket weaving using reeds. Carpentry. Matowe - African chewing gum. Roofing and thatching huts. Pottery. Traditional dyes (for painting clothing materials and other objects).*

### How relevant is the indigenous Chemistry knowledge to Chemistry metacognition?

Pre-service science teachers were of the opinion that Indigenous Chemistry knowledge has the same Chemistry ideas/concepts which are found in the western Chemistry knowledge. The indigenous Chemistry knowledge is the Chemistry prior knowledge for western Chemistry knowledge. Indigenous Chemistry knowledge and skills have numerous beneficial characteristics in most aspects of life in general. However, it is their invaluable assistance in understanding Chemistry concepts which was also mentioned by pre-service science teachers among other benefits in terms of Chemistry metacognition. The fact that they are passed from one generation to the next generation ensures continuity of chemistry prior knowledge which is a vital bridge between indigenous chemistry knowledge and western Chemistry knowledge. Pre-service science teachers were also in agreement that the indigenous Chemistry knowledge and skills are very efficient.

Indigenous Chemistry knowledge and skills are seen as useful and reliable by pre-service science teachers to a larger extent. However, the pre-service science teachers identified the aspect of some of the indigenous Chemistry knowledge and skills as being seasonal that can pose challenges on reliability. The experiences of indigenous Chemistry knowledge and skills that have been had by the majority of the pre-service science teachers are positive. Having positive experiences with indigenous Chemistry knowledge and skills means the pre-service science teachers will less easily forget the acquired indigenous Chemistry knowledge and skills. They went on to say these indigenous Chemistry knowledge and skills are relatively easy to master, though it depends on the complexity of the indigenous Chemistry knowledge and skills to be mastered. This implies that the majority of pre-service science teachers have acquired some indigenous Chemistry knowledge, skills and attitudes from their everyday cultural experiences.

There are some common characteristics between indigenous Chemistry knowledge and western Chemistry knowledge that were identified by pre-service science teachers. These included taking precautions when using them, procedures and processes to be followed, formal chemistry is based on indigenous chemistry and serve the same purpose of providing chemical solutions to chemical challenges. Pre-service science teachers are aware that there is a relationship between indigenous Chemistry knowledge and formal Chemistry with the indigenous Chemistry knowledge being the known (prior chemistry knowledge) and college Chemistry the unknown in chemistry teaching and learning.

### How effective is the indigenous Chemistry knowledge in Chemistry metacognition?

**Figure 2.**
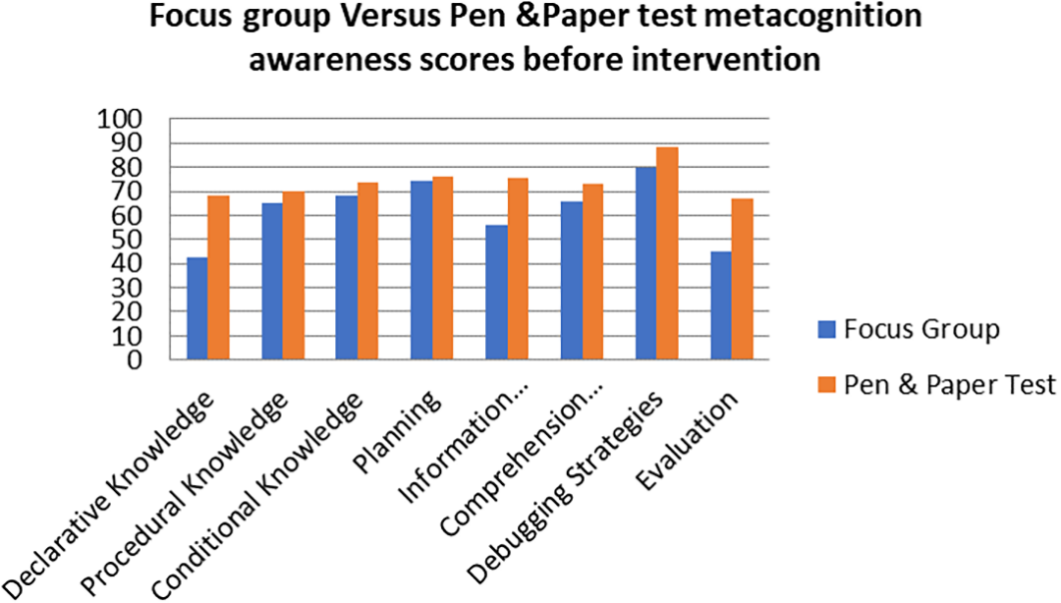
Comparison between focus group with pen & paper test.

A comparison of the focus group with pen and paper test metacognition awareness scores before intervention for the pre-service science teachers are shown in Figure 2.

The pen and paper test metacognition awareness scores are higher than for focus group metacognition awareness scores before the intervention. The Pearson product moment correlation was used to correlate the focus group metacognition awareness scores with those of the pen and paper test metacognition awareness scores before the indigenous Chemistry knowledge intervention. The correlation was found to be 0.79 which shows a strong positive correlation between the focus group metacognition awareness scores and pen and paper test metacognition awareness scores before intervetion. The variance of the focus group metacognitive awareness scores that is accounted for by the variance in the pen and paper test metacognition awareness scores is 62% before the intervetion.

**Figure 3.**
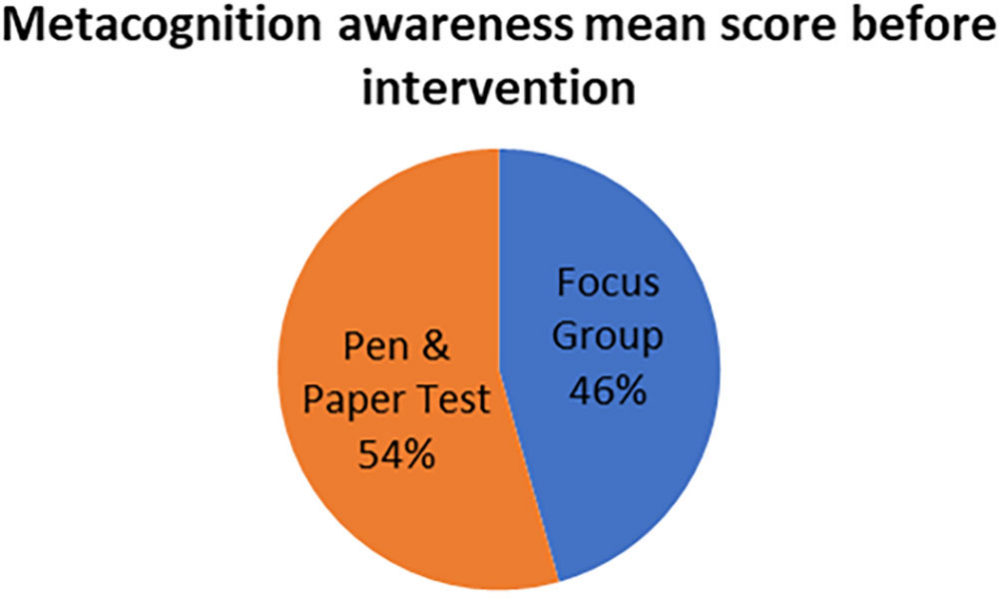
Pre-intervention test versus interview metacognition awareness.

The mean scores for focus group metacognition awareness as well as for pen and paper test metacognition awareness before intervention are indicated in Figure 3.

There is a slight difference in the mean scores implying a slight difference in metacognition awareness between focus group and pen and paper test before intervention. This indicates that the results of the focus group metacognition awareness are to a large extent confirmed by the pen and paper metacognition awareness test in terms of triangulation.

In the observations for the application of indigenous chemistry knowledge, the application of indigenous Chemistry knowledge in the lectures before the intervention phase was rare except for second lecture where it was medium standard. This was when the “Plum pudding” model of the atom was described using a positively charged pumpkin, which has pumpkin seeds acting as the electrons by one pre-service science teacher. However, after metacognition instruction using indigenous Chemistry knowledge as prior knowledge, there was a high standard of the application of indigenous Chemistry knowledge in Chemistry concepts. Also, in the two Chemistry practical assessments that were done, the application of indigenous Chemistry knowledge was high though most of the time negatively. This suggests that the indigenous Chemistry knowledge intervention was effective as pre-service science’s frequency of using indigenous Chemistry in the Chemistry lectures had improved.

**Figure 4.**
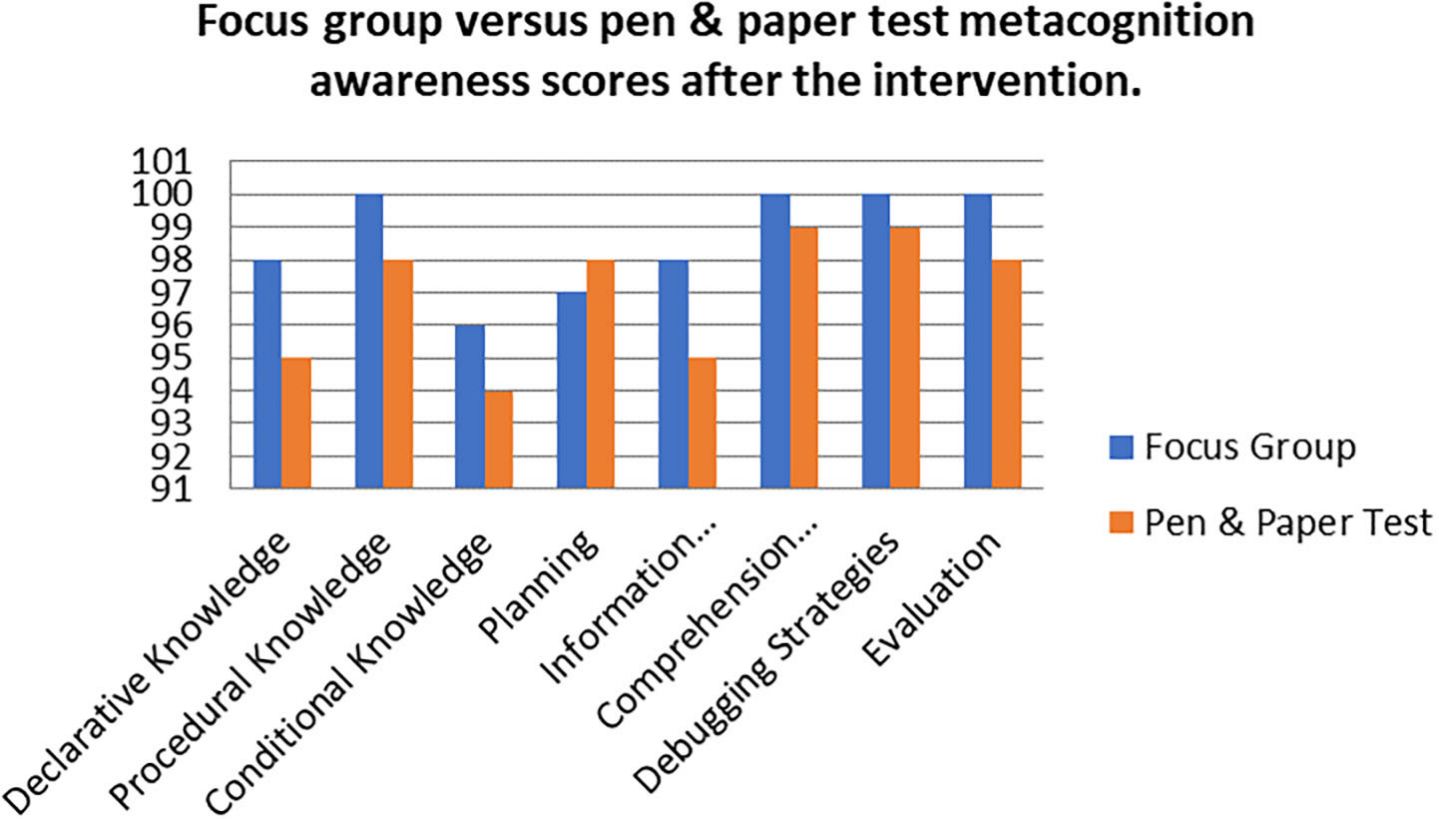
Post intervention comparison between interview and test scores.

A comparison of the focus group with pen and paper test metacognition awareness scores after intervention for the pre-service science teachers are shown in Figure 4.

There was a high metacognition awareness for the focus groups when compared to pen and paper test in most of the metacognition components except for planning after the indigenous Chemistry knowledge intervention. This is the opposite for the scores before the indigenous Chemistry knowledge intervention in which the metacognition awareness for focus groups was lower when compared to the pen and paper test. There is no consistency in the increase of the scores.

The Pearson product moment correlation was used to correlate the focus group metacognition awareness scores with the pen and paper test metacognition awareness scores after the indigenous Chemistry knowledge intervention. The correlation was found to be 0.76 which shows a strong positive correlation between the focus group metacognition awareness scores when correlated to the pen and paper test metacognition awareness scores after the intervention. The variance of the focus group metacognitive awareness scores that is accounted for by the variance in the pen and paper test metacognition awareness scores is 58% after the intervention.

**Figure 5.**
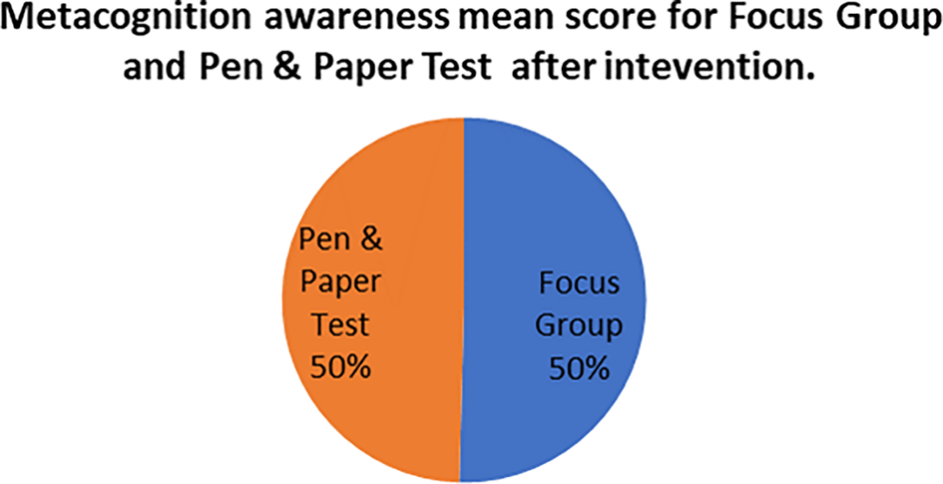
Metacognition awareness mean scores after the intervention.

The mean score of focus groups when compared to the mean score of the pen and paper tests after the indigenous Chemistry knowledge intervention give the extent to which the two are in agreement. The mean scores for focus group metacognition awareness as well as for pen and paper test metacognition awareness before the indigenous Chemistry knowledge intervention are indicated in Figure 5.

**Figure 6.**
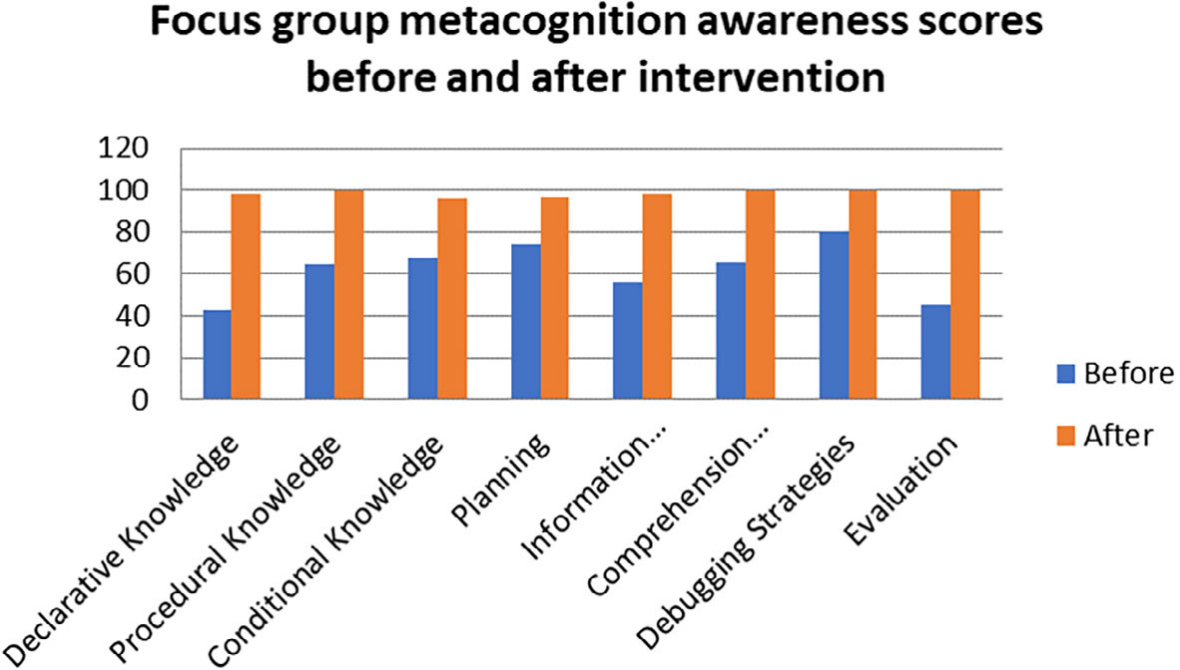
Comparison of focus group scores before and after intervention.

There was no difference in the mean scores implying there was no difference in the metacognition awareness after the indigenous Chemistry knowledge intervention of the mean score of the focus groups when compared to the mean score of the pen and paper test after the indigenous Chemistry knowledge intervention. This indicates that the results of the focus group metacognition awareness were confirmed by the paper and pen metacognition awareness test after the indigenous Chemistry knowledge intervention in terms of triangulation. The impact of the indigenous Chemistry knowledge intervention on metacognitive awareness can be ascertained by comparing the focus group scores after the indigenous Chemistry knowledge intervention to those before the indigenous Chemistry knowledge intervention. A comparison of the scores after the indigenous Chemistry knowledge intervention and before the indigenous Chemistry knowledge intervention are shown in Figure 6.

**Figure 7.**
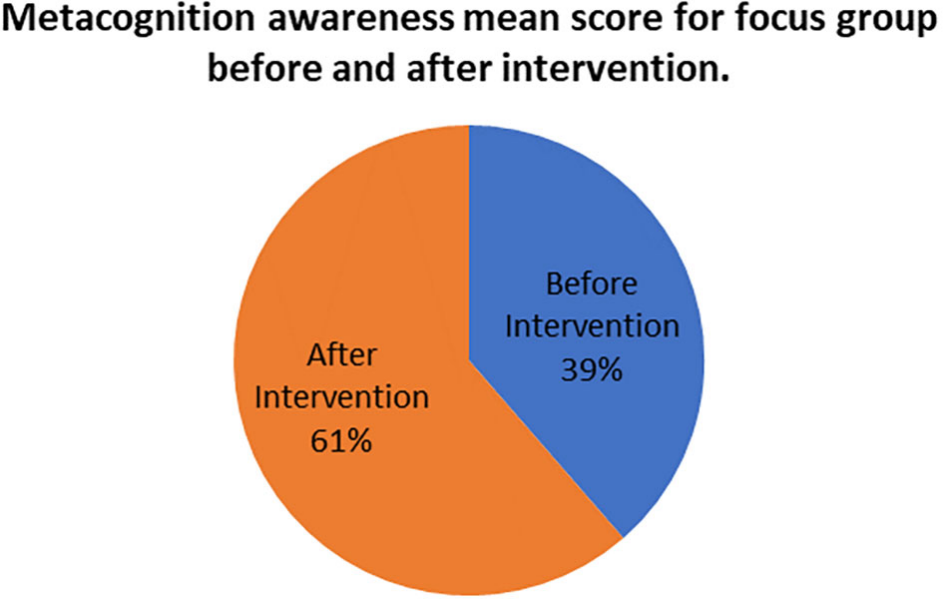
Metacognition awareness mean scores before and after intervention.

The metacognition awareness scores are higher after the indigenous Chemistry knowledge intervention when compared to the metacognition awareness before the indigenous Chemistry knowledge intervention. The indigenous Chemistry knowledge intervention had an effect in the positive direction on the metacognition awareness. The indigenous Chemistry knowledge intervention had an effect of improving the metacognition awareness of the pre-service science teachers. Comparing the means scores of the focus group before and after the indigenous Chemistry knowledge intervention gives an idea of the degree of change after the indigenous Chemistry knowledge intervention. Figure 7 shows this comparison.

**Figure 8.**
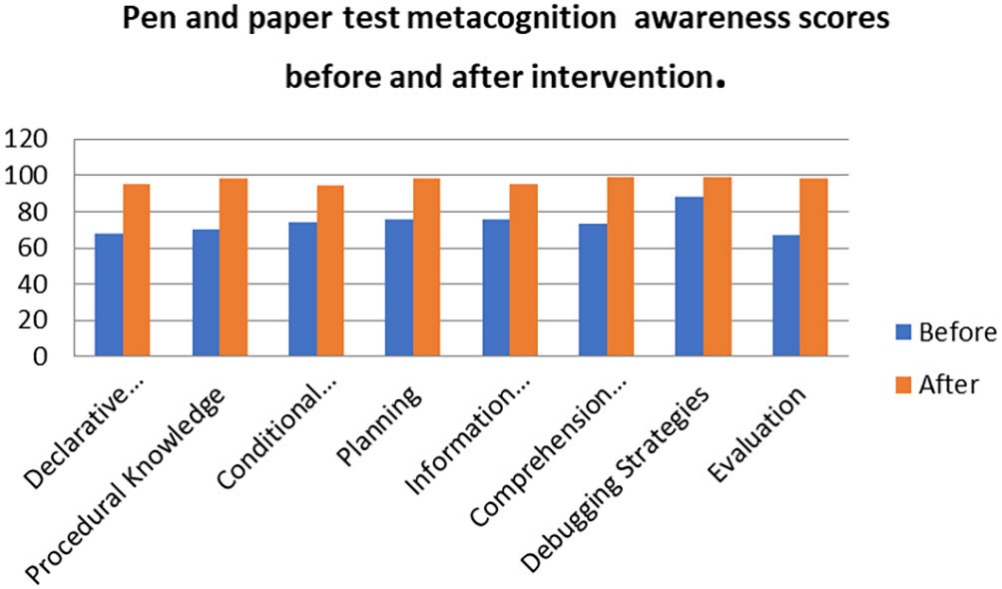
Comparison of pen and paper metacognition awareness scores.

There is a sharp difference between the focus group metacognition awareness mean score before the indigenous Chemistry knowledge intervention and focus group mean score after the indigenous Chemistry knowledge intervention. The metacognition awareness mean score of the focus groups increased after the indigenous Chemistry knowledge intervention. This implies a sharp increase in metacognition awareness as a result of the indigenous Chemistry knowledge intervention. The impact of the indigenous Chemistry knowledge intervention can be found by comparing the pen and paper metacognition awareness scores after the indigenous Chemistry knowledge intervention to those before intervention. Figure 8 shows a comparison of the pen and paper metacognition awareness scores after the indigenous Chemistry knowledge intervention to those before the intervention.

**Figure 9.**
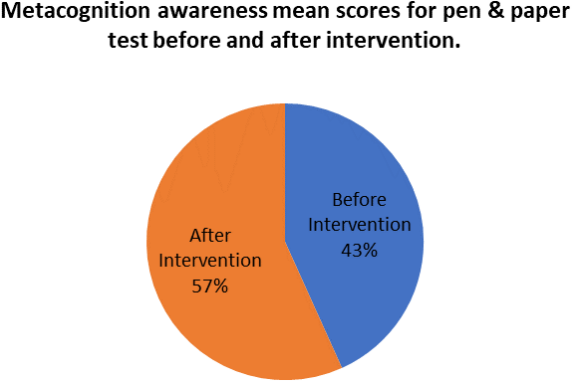
Metacognition awareness mean score before and after intervention.

The pen and paper test metacognition awareness scores are higher after the indigenous Chemistry knowledge intervention. The indigenous Chemistry knowledge intervention had an effect of increasing the metacognition awareness. There is a consistency in the increase in metacognition awareness for both the focus groups and pen and paper test after the indigenous Chemistry knowledge intervention. A comparison of the pen and paper test metacognitive awareness mean scores before and after the indigenous Chemistry knowledge intervention shows changes that might have occurred as a result of the intervention. Figure 9 shows a comparison of pen and paper metacognitive awareness scores before and after intervention for the pre-service science teachers.

**Figure 10.**
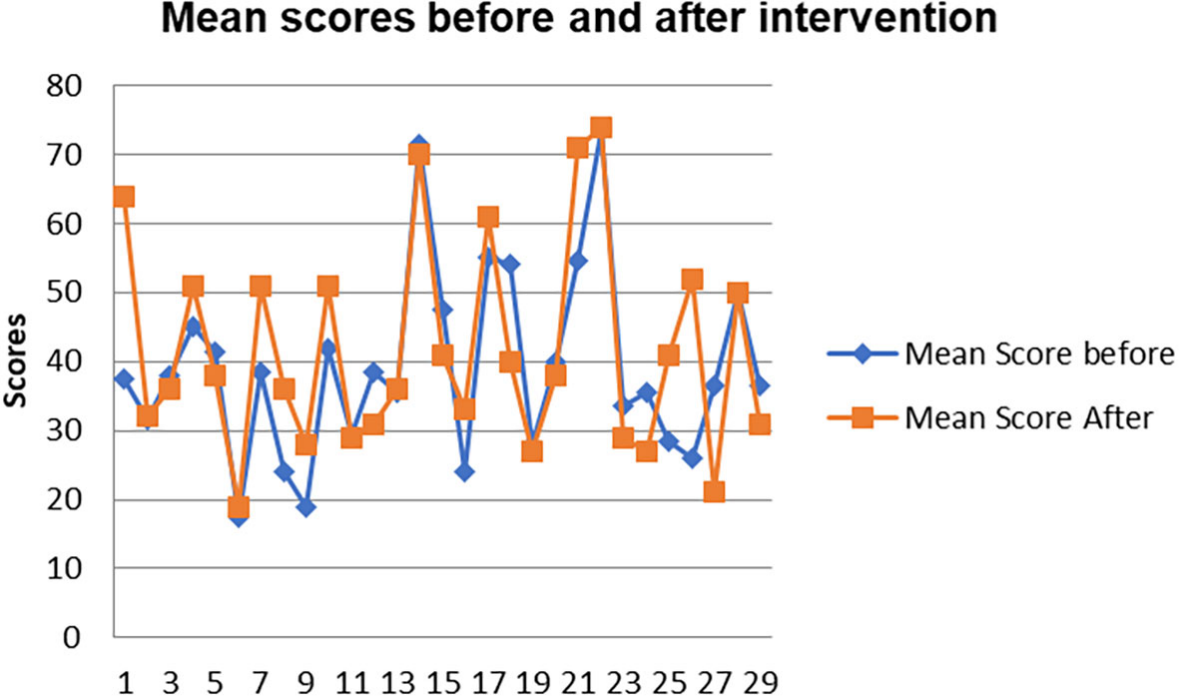
Comparison of means scores before and after intervention.

There is a significant difference in metacognition awareness mean scores before and after the indigenous Chemistry knowledge intervention. The metacognition awareness mean score of the pen and paper test increased after the indigenous Chemistry knowledge intervention. The implication is that there is a significant increase in metacognition awareness as a result of the indigenous Chemistry knowledge intervention. The change in academic performance of the pre-service science teachers can be identified by the differences in the mean scores of assignments and tests before and after the indigenous Chemistry knowledge intervention. The mean scores for assignments and tests before and after the indigenous Chemistry knowledge intervention for pre-service science teachers are shown in Figure 10.

The pre-service science teachers’ mean scores of the Chemistry assignment one and chemistry test one before indigenous Chemistry knowledge intervention are lower than the mean scores of the assignment two and test two & three after intervention. This implies that the academic performance of the pre-service science teachers improved after the indigenous Chemistry knowledge intervention.

## Discussion

The purpose of this embedded mixed methods case study was to explore the influence of indigenous Chemistry knowledge on Chemistry metacognition. This was achieved through interrogating; the indigenous Chemistry knowledge held by the respondents, the relevance and effectiveness of indigenous Chemistry knowledge to Chemistry metacognition. The sample size was a limiting factor even though in depth analysis of data was of paramount importance as the findings could relate to many context similar to this one.

The findings suggest that pre-service science teachers have a wealth of indigenous Chemistry knowledge in a variety of fields, including agriculture, environmental conservation, food processing, food preservation, and health care. The conclusions of this study are consistent with Mapara’s (2009) findings, which showed that local people still possess indigenous knowledge despite years of colonization. This knowledge includes fields like zoology, botany, agriculture, medicine, and artisan skills. These findings are in line with those of Ugboma (2014), whose research indicated that most people have access to and use indigenous knowledge. According to Senanayake’s (2006) research, experts in indigenous knowledge are typically members of society.

The results suggest a relevance of indigenous Chemistry knowledge to Chemistry metacognition as it is the prior knowledge for Chemistry metacognition. Metacognition requires a learner to have prior knowledge in order for one to learn it. Prior knowledge is required for the development of metacognitive skills as it facilitates and assist in the development of the cognitive skills states Alshammari (2015). The Chemistry concepts in indigenous Chemistry knowledge are the same as those found in western Chemistry knowledge, hence their relevance to Chemistry metacognition. As a result of this relevancy, there are calls for the integration of indigenous knowledge into science education.

Aspects of culture of non-western science learners such as indigenous knowledge (traditional and empirical knowledge), ways of knowing as well as indigenous world views should be taken into account and incorporated as science learning foundations ( Quigley, 2009). This is in agreement with Ugwu and Diovu (2016), who opine that indigenous knowledge and their practices’ integration into the teaching of chemistry enhances learners’ Chemistry understanding and achievement. Another finding was that there are many similarities between indigenous Chemistry knowledge and western (college) Chemistry knowledge that exist. Tsuji and Ho (2002) emphasised the importance and benefits of identifying similarities between indigenous Chemistry knowledge and western Chemistry knowledge ways of knowledge construction and transmission so as to enhance understanding of the natural world.

Results from this study imply that indigenous Chemistry knowledge is quite effective in improving Chemistry metacognition as there was an increase in terms of metacognition awareness after the indigenous Chemistry knowledge intervention. This is in agreement with Schraw (1998), who identified four ways of classroom metacognition awareness promotion. These are: highlighting metacognition importance, knowledge of cognition improvement, regulation of cognition improvement and metacognitive awareness environment fostering. This is a new insight into the relationship between indigenous Chemistry knowledge and Chemistry metacognition which has never been studied before. According to Schraw (2000), the reliability of the Metacognitive Awareness Inventory (MAI) in measuring metacognition is extremely high. A study by Van Aswegen (2015) focused on using stories to develop the metacognitive awareness of intermediate phase young learners in South Africa. Most studies have focused on the impact of metacognition on academic performance.

## Conclusions and implications

From the findings, the conclusion drawn is that Chemistry educators such as teachers and lecturers have access to indigenous Chemistry knowledge that is held and practiced by Chemistry learners in their everyday lives for survival. This indigenous Chemistry knowledge represents alternative Chemistry concepts or Chemistry misconceptions from the Chemistry learners’ social-cultural life which can either promote or disrupt the western Chemistry teaching and learning process. It is recommended that the indigenous Chemistry knowledge of chemistry learners should be identified and applied constructively in the Chemistry curriculum at teachers’ colleges, thereby contextualising the western Chemistry education.

The findings suggest that indigenous Chemistry knowledge is relevant to chemistry metacognition as it is the prior knowledge for Chemistry metacognition since it utilises empirical Chemistry ideas and concepts. As a result, the learners’ indigenous Chemistry knowledge assists in the understanding of western Chemistry concepts as it comes from learners’ everyday socio-cultural life experiences. It can be concluded that Chemistry metacognition can be successfully taught or increased in Chemistry learners by applying indigenous Chemistry knowledge in Chemistry education. What is recommended is that Chemistry educators should be capacitated with the knowledge and skills for identifying and applying learners’ indigenous Chemistry knowledge that is relevant to Chemistry metacognition.

The evidence suggests that indigenous Chemistry knowledge is quite effective in influencing Chemistry metacognition positively as there was an improvement of metacognition awareness after the intervention. The evidence also suggests that the intervention increased the academic performance of the pre-service science teachers in Chemistry. The conclusion drawn from the evidence is that Chemistry metacognition and the academic performance of Chemistry learners are significantly influenced by the use of indigenous Chemistry knowledge. The recommendation is that a deliberate systemic conscientisation of the importance of using indigenous Chemistry knowledge in Chemistry education for the purposes of Chemistry metacognition should be made to Chemistry learners and educators as well as other stakeholders.

### The process of chemistry metacognition

**Figure 11.**
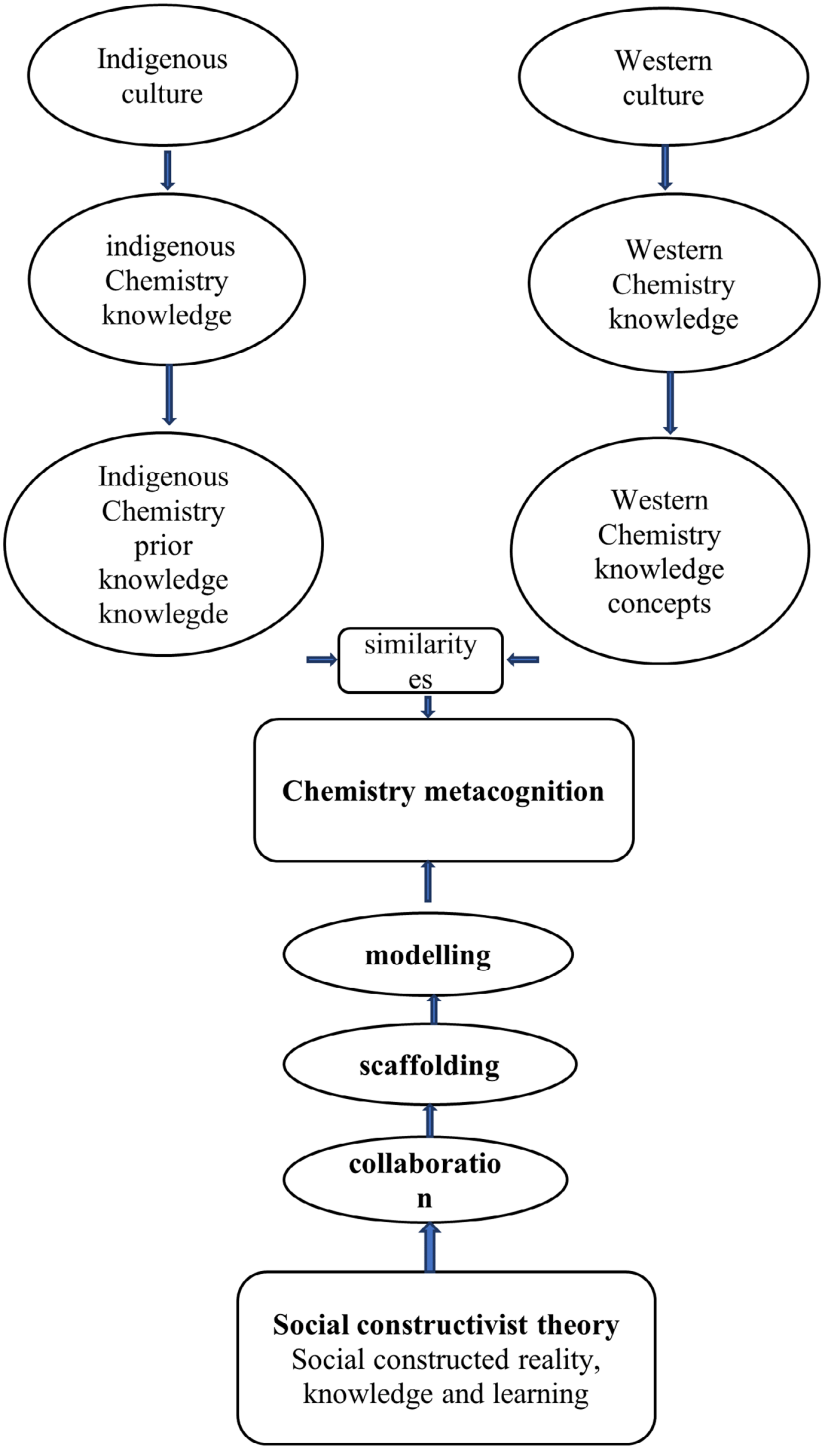
The process of chemistry metacognition.

Chemistry metacognition is the recognition of the value of one’s extensive and systematic native Chemistry knowledge (prior knowledge) with an accurate assessment of the demands of a challenging western Chemistry learning activity, what understanding and skills are needed, as well as the intelligence required to make the appropriate deduction on how to apply one’s native Chemistry knowledge reliably and effectively in a particular situation. The study has shown how the process of chemistry metacognition occurs as indicated in Figure 11.

### Major contributions of the study to chemistry education literature

**Figure 12.**
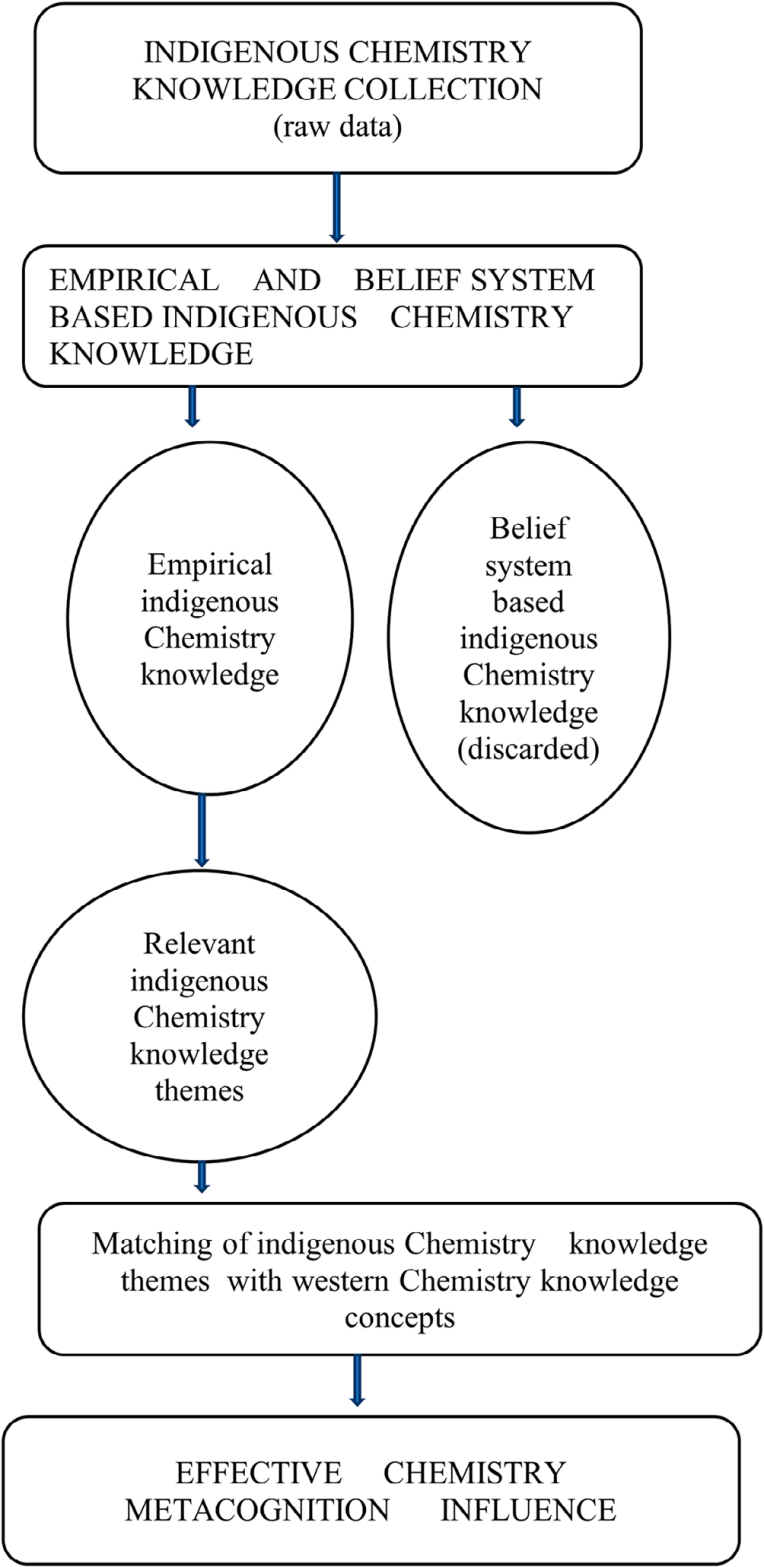
Contributions of study to chemistry education.

Four important major contributions to the literature on the influence of indigenous Chemistry knowledge on Chemistry metacognition have been made by this study due to the research in these four areas being relatively new and there is still limited related literature in this area. Figure 12 shows the major contributions of the study.

First, the research participants were first year post ordinary level science pre-service science teachers who had no experience of Chemistry education at a tertiary level and found the Chemistry to be challenging, particularly for those who had no previous Chemistry education at any level of education. This study should contribute to the development of methods of identifying and collecting ideological, rational, communal and empirical indigenous Chemistry knowledge possessed by Chemistry learners for use in Chemistry education at this level. The sacred nature of some of the indigenous Chemistry knowledge as well as its visual or oral forms of transmission might pose challenges in terms of its accurate collection for use in Chemistry education.

Second, the study looked at the relevance of indigenous Chemistry knowledge in Chemistry metacognition research of which is almost non-existent when compared to the research on the effect of metacognition on academic performance ( Van Aswegen, 2015; Aurah, Koloi-Keaikitse, Isaacs and Finch, 2011; Rahman, Jumani, Chaudry, Chitsti and Abbasi, 2010). The findings of this study should enhance the literature on the relationship between indigenous Chemistry knowledge and Chemistry metacognition, particularly on how the former influences the latter.

Third, the effectiveness of indigenous Chemistry knowledge on Chemistry metacognition was looked at in this study, which is not familiar to most Chemistry educators. The findings of this study might attract other Chemistry educators to the effect of indigenous Chemistry knowledge on Chemistry metacognition. There is more in-depth Chemistry learning and improved academic performance by Chemistry learners who have acquired Chemistry metacognition skills. Finally, this study on the influence of indigenous Chemistry knowledge on Chemistry metacognition improved the originality of the research. Although the impact of metacognition on academic performance has been analysed since the 1970s ( Akman and Alagoz, 2018), the influence of indigenous Chemistry knowledge on Chemistry metacognition has never been done before. This study therefore contributes to new literature in this area.

## Data availability

### Underlying data

The raw responses to the questionnaire cannot be shared as they cannot be effectively deidentifed. These data can be obtained directly from Dr Tavonga Tawanda at tavongatawanda@gmail.com.

Figshare. The influence of indigenous knowledge on chemistry metacognition. https://doi.org/10.6084/m9.figshare.22666102.v1 ( Mudau, 2023a).

The project contains the following underlying data:

• Metacognition awareness asseesment before and after.docx
• Phd assignment and test scores.docx
• Unisa 2020 focus group demographic information.doc
• Unisa 2020 group five indigenous chemistry knowledge focus group feedback.docx
• Unisa 2020 group four indigenous chemistry knowledge group feedback.docx
• Unisa 2020 group one indigenous chemistry knowledge focus group feedback.docx
• Unisa 2020 group three indigenous chemistry knowledge focus group feedback.docx
• Unisa 2020 group two indigenous chemistry knowledge focus group feedback.docx

Figshare: Metacognition awareness assessment.xlsx. https://doi.org/10.6084/m9.figshare.22761347.v1 ( Mudau, 2023b).

### Extended data

Figshare: The influence of indigenous knowledge on chemistry metacognition. https://doi.org/10.6084/m9.figshare.22666135.v1 ( Mudau, 2023c).

This project contains the following extended data:

• Application of indigenous chemistry knowledge observation schedule.doc
• Indigenous chemistry knowledge focus group questions.doc
• Metacognition awareness focus group questions.doc
• Metacognition awareness paper and pen test.doc

### Reporting guidelines

Figshare. Completed SRQR checklist for “The influence of indigenous knowledge on chemistry metacognition’ https://doi.org/10.6084/m9.figshare.22666153.v1 ( Mudau, 2023d).

Data are available under the terms of the Creative Commons Attribution 4.0 International license (CC-BY 4.0).
