# Supplementary material for: The influence of indigenous knowledge on chemistry metacognition
Source: F1000Res. 2024 Feb 29;12:589. Originally published 2023 Jun 1. [Version 4] doi: 10.12688/f1000research.131685.4 (PMC11109572; doi:10.12688/f1000research.131685.4)
Supplement: Peer review history

# Reviewer report: Reviewer response for version 4

**DOI:** 10.5256/f1000research.163028.r260063

**Published:** 2024-05-09

**Authors:** Samuel Ouma Oyoo

**Affiliations:** 1 Maseno University, Maseno, Kisumu, Kenya

This article is a report of an investigation of the influence of indigenous knowledge on chemistry metacognition. The idea for the study reported here is innovative, the design is appropriate, the technical issues about the study are fine. It is a good report as it stands. The findings are directly useful to chemistry educators in the context of the study and beyond. What the authors need to, however, relook includes the following:

 1. Several typographical errors and omissions have been detected along the article and these if eliminated will give the article a better flow.   This calls for a closer proofreading of the work.

 2. The structure of the article can be reconsidered: a) Table 1 - 6 in the results section are the heart of the study and could be moved to the Appendix. b) The results section need be signposted more explicitly. As currently presented, it is one long read, with the reader left to self-guide along the section. 

 Overall, however, this is a timely contribution to knowledge, both in science education and science teacher preparation.

Is the work clearly and accurately presented and does it cite the current literature?

Yes

If applicable, is the statistical analysis and its interpretation appropriate?

Yes

Are all the source data underlying the results available to ensure full reproducibility?

No source data required

Is the study design appropriate and is the work technically sound?

Yes

Are the conclusions drawn adequately supported by the results?

Yes

Are sufficient details of methods and analysis provided to allow replication by others?

Yes

Reviewer Expertise:

Science education, science teacher education, language use in/for effective teaching and learning of science.

I confirm that I have read this submission and believe that I have an appropriate level of expertise to confirm that it is of an acceptable scientific standard.

# Reviewer report: Reviewer response for version 3

**DOI:** 10.5256/f1000research.159538.r230732

**Published:** 2024-02-15

**Authors:** Nadaraj Govender

**Affiliations:** 1 School of Education, University of KwaZulu-Natal, Durban, KwaZulu-Natal, South Africa

The paper is well strengthened. Do check the figures as some words in the blocks run to the next line.

Is the work clearly and accurately presented and does it cite the current literature?

Yes

If applicable, is the statistical analysis and its interpretation appropriate?

I cannot comment. A qualified statistician is required.

Are all the source data underlying the results available to ensure full reproducibility?

Yes

Is the study design appropriate and is the work technically sound?

Yes

Are the conclusions drawn adequately supported by the results?

Yes

Are sufficient details of methods and analysis provided to allow replication by others?

Yes

Reviewer Expertise:

Science and Indigenous Knowledge Education

I confirm that I have read this submission and believe that I have an appropriate level of expertise to confirm that it is of an acceptable scientific standard.

# Reviewer report: Reviewer response for version 3

**DOI:** 10.5256/f1000research.159538.r230733

**Published:** 2024-01-30

**Authors:** Maria Tsakeni

**Affiliations:** 1 Faculty of Education, University of the Free State, Bloemfontein, Free State, South Africa

While most of the issues raised in the previous review have been explained or addressed, I still think the authors have to add the perspective of IKS that they used. I understand the explanation that metacognition is the theoretical/conceptual framework selected for the study. However, the study also addresses a very significant concept, IKS, for which there is a wide array of literature leading to varied perspectives. Adding a paragraph or two to address this issue may improve the manuscript.

Is the work clearly and accurately presented and does it cite the current literature?

Partly

If applicable, is the statistical analysis and its interpretation appropriate?

Not applicable

Are all the source data underlying the results available to ensure full reproducibility?

Yes

Is the study design appropriate and is the work technically sound?

Yes

Are the conclusions drawn adequately supported by the results?

Yes

Are sufficient details of methods and analysis provided to allow replication by others?

Partly

Reviewer Expertise:

Chemistry education, Research in innovative instructional strategies and curriculum innovations in science classrooms.

I confirm that I have read this submission and believe that I have an appropriate level of expertise to confirm that it is of an acceptable scientific standard, however I have significant reservations, as outlined above.

# Response

**Published:** 2024-02-22

**Authors:** Awelani Mudau

**Affiliations:** Science and Technology Educati, University of South Africa, Pretoria, Gauteng, South Africa

All the suggestions made by the reviewer have been checked again and addressed. We apologize for missing some of them. it is not a good thing but we are content that this time they have been addressed

# Reviewer report: Reviewer response for version 2

**DOI:** 10.5256/f1000research.158043.r220265

**Published:** 2023-11-21

**Authors:** Nadaraj Govender

**Affiliations:** 1 School of Education, University of KwaZulu-Natal, Durban, KwaZulu-Natal, South Africa

The study is very interesting and contributes to the field. Please see some concerns in the attached annotated pdf. There are some claims made but not substantiated by evidence in this study. Also some clarity is needed.

 A critical perspective of IK is also needed so as not to romantize  it as the panacea for quick solutions regarding  higher performance as concepts in Chemistry is largely embedded in the history/lab work/theorization of chemistry  from the West but contexts and examples in Africa can scaffold learning which the paper attempts to do.

Is the work clearly and accurately presented and does it cite the current literature?

Yes

If applicable, is the statistical analysis and its interpretation appropriate?

I cannot comment. A qualified statistician is required.

Are all the source data underlying the results available to ensure full reproducibility?

Yes

Is the study design appropriate and is the work technically sound?

Yes

Are the conclusions drawn adequately supported by the results?

Yes

Are sufficient details of methods and analysis provided to allow replication by others?

Yes

Reviewer Expertise:

NA

I confirm that I have read this submission and believe that I have an appropriate level of expertise to confirm that it is of an acceptable scientific standard, however I have significant reservations, as outlined above.

# Response

**Published:** 2023-11-29

**Authors:** Awelani Mudau

**Affiliations:** Science and Technology Educati, University of South Africa, Pretoria, Gauteng, South Africa

**REVIEWER SUGGESTIONS**

**CHANGES MADE**

 The paragraph on components of metacognition needs some clarity as confusing-maybe a block diagram

 A block diagram of metacognition and its components as figure 1 have been added

 ,that is, procedural

 that is, procedural

 This paragraph be part of previous paragraph again in the same block diagram will help

 The paragraph has been made part of the previous diagram and same block diagram

 This sentence is confusing… encouraged by...

 The sentence is “Metacognition promotes independent Chemistry knowledge, knowledge that is more permanent, motivates learners and improves educational achievement across different ages, intellectual abilities and subject areas”

 Is this in general or in Zimbabwe specifically

 It is in Zimbabwe and also in general

 Sentence not clear… why learners now?

 Corrected to August Vacation School from August Vacation College. Learners are the ones who were attending the August Vacation School. These are high school learners who enroll as pre-service science teachers at the teachers’ college.

 How do we know this is ideal? Where’s the evidence-- this is an opinion then needed to stated a such.

 Authorities that show that this is ideal have been added. Oladejo,Okebukola,Olateju,Akinola, Ebisinand Dansu(2022);Verharen, Gutema,Tharakan,Bugarin,Fortunak, Kadoda,Liu, and Middendorf, 2014);Buchanan, 2012).

 Cannot draw this No evidence of academic performance.

 This can be drawn as figure 10 shows the evidence of academic performance

**References**

 Oladejo,A.I., Okebukola,P.A.,Olateju, T.T,Akinola, V.O., Ebisin,A  and Dansu, T.V. In search of culturally responsive tools for meaningful learning of chemistry in Africa: we stumbled on the culturo-techno- contextual approach. *J. Chem. Educ.2022 *, 99 (8) 2919–2931

https://doi.org/10.1021/acs.jchemed.2c00126

Buchanan, M.S.‘Drugs, religion and chemistry in Tanzania’: an interactive seminar for chemistry students.The Royal Society of Chemistry: Chemistry Education Research and Practice. 2012. 1(9):1-9. DOI: 10.1039/x0xx00000x

Verharen,C., Gutema,B., Tharakan,T. Bugarin,F.,Fortunak,J Kadoda,G.,Liu,M and Middendorf,G. African philosophy: a key to African innovation and development. African Journal of Science, Technology, Innovation and Development. 2014. 6(1):3-12, DOI:10.1080/20421338.2014.902565

# Reviewer report: Reviewer response for version 1

**DOI:** 10.5256/f1000research.144552.r185591

**Published:** 2023-07-25

**Authors:** Nadaraj Govender

**Affiliations:** 1 School of Education, University of KwaZulu-Natal, Durban, KwaZulu-Natal, South Africa

1. The area of research is unique and new to the field in indigenous knowledge connected to metacognition and makes a distinct contribution to education.
  However the literature and research focus must focus on Preservice teachers (PST) mostly and not on learners at school as the sample are PSTS and research questions based on PST. [ see comments in doc]
2. The concept of metacognition is very briefly discussed in the beginning. This must be elaborated in depth as it is the KEY concept in the study. Do link with updated and well cited articles in chemistry education.
3. As Table 1-6 is a list of areas of IK, I think it’s not bringing out the experiences of the students and hence the data seems like it is a literature search. Possibly do add interviews on some aspects from Tables 1-6 which will add authenticity to the data.
4. There are lots of spelling errors. Suggest a thorough edit and check. [ see doc]

Is the work clearly and accurately presented and does it cite the current literature?

Yes

If applicable, is the statistical analysis and its interpretation appropriate?

I cannot comment. A qualified statistician is required.

Are all the source data underlying the results available to ensure full reproducibility?

Yes

Is the study design appropriate and is the work technically sound?

Yes

Are the conclusions drawn adequately supported by the results?

Yes

Are sufficient details of methods and analysis provided to allow replication by others?

Yes

Reviewer Expertise:

Science and Indigenous Knowledge Education

I confirm that I have read this submission and believe that I have an appropriate level of expertise to confirm that it is of an acceptable scientific standard, however I have significant reservations, as outlined above.

# Response

**Published:** 2023-09-06

**Authors:** Awelani Mudau

**Affiliations:** Science and Technology Educati, University of South Africa, Pretoria, Gauteng, South Africa

**1. Is the work clearly and accurately presented and does it cite the current literature? Partly**

 You have raised an important point here. The “partly” aspect is not clear on what it refers to exactly in this comment as there is clear and accurate as well as citing current literature in the comment. However, we are of the opinion that the presentation of the work was clear and accurate as it focused on the influence of indigenous knowledge on chemistry metacognition from, introduction, research questions, literture review, research methods, results and conclusion are clear and accurate as well as current literature was used at the time of the study

 2. **Is the study design appropriate and is the work technically sound?**Yes

 Thank you for pointing this out. We concur with your comment

 3. **Are sufficient details of methods and analysis provided to allow replication by others?**Partly

 You have raised an important point here. However, we are of the opinion that in the context of the study, the methods and analysis are sufficient for replication by others as they fully answered the research questions and met the  the research objectives

 4. **If applicable, is the statistical analysis and its interpretation appropriate?**Not applicable

 We take note of your view. However, we are of the opinion that the statistical analysis and interpretation is appropriate and applicable for this study as it gives the scientific degree to which the focus group and paper and test metacognition awareness scores are in agreement in terms of triangulation

 5. **Are all the source data underlying the results available to ensure full reproducibility?**Yes

 Thank you for pointing this out. We concur with your comment

 6. **Are the conclusions drawn adequately supported by the results?**Yes

       Thank you for pointing this out. We concur with  your comment

# Reviewer report: Reviewer response for version 1

**DOI:** 10.5256/f1000research.144552.r176497

**Published:** 2023-06-19

**Authors:** Maria Tsakeni

**Affiliations:** 1 Faculty of Education, University of the Free State, Bloemfontein, Free State, South Africa

I think this study is important because it opens up opportunities to teach chemistry in context using IKS. Mixed methods were used. I think research question 1, What is the indigenous Chemistry knowledge possessed by pre-service science teachers? was addressed because the indigenous knowledge systems possessed by the students were identified. The insight out of the second research question, How relevant is the indigenous Chemistry knowledge to Chemistry metacognition? was that Indigenous chemistry serves as prior knowledge in chemistry classrooms.

 I however thought that if the authors had selected an IKS conceptual framework to use in the study they were going to be able to unpack this insight more deeply. The insights may help us to clarify the understanding of IKS or indigenous chemistry in this case, that it is in fact applied chemistry and that the two types of knowledge are not different from each other.

 I also have an issue with branding some of the identified knowledge/practices as non-western. Let's take just one example of tree planting to avoid soil erosion. I think that is a universal agricultural practice in the west in Africa and everywhere. This is why I say it would have been good to have a conceptual framework that shows the the overlaps and differences in any of the two knowledge systems. The research question is however addressed.

 The third research question, How effective is the indigenous Chemistry knowledge in Chemistry metacognition? pertained to the quantitative methods used. My question is how comparisons were made between data from focus group interviews (which I assume to be qualitative) and data from papers tests (whose scores can be used as quantitative data). Until the authors explain how they got quantitative data from focus group interviews to be able to calculate correlations, this part is difficult to accept as valid. There is an need to look at the methods again and provide the missing information. For example it is not sufficient to say data were analyzed through SPSS version 23 without specifying the particular comparison tool used.

Is the work clearly and accurately presented and does it cite the current literature?

Partly

If applicable, is the statistical analysis and its interpretation appropriate?

Not applicable

Are all the source data underlying the results available to ensure full reproducibility?

Yes

Is the study design appropriate and is the work technically sound?

Yes

Are the conclusions drawn adequately supported by the results?

Yes

Are sufficient details of methods and analysis provided to allow replication by others?

Partly

Reviewer Expertise:

Innovative instructional strategies such as inquiry-based learning, practical work, instructional technologies and curriculum innovations such as STEM education, IKS and ESD in science classrooms

I confirm that I have read this submission and believe that I have an appropriate level of expertise to confirm that it is of an acceptable scientific standard, however I have significant reservations, as outlined above.

# Response

**Published:** 2023-09-04

**Authors:** Awelani Mudau

**Affiliations:** Science and Technology Educati, University of South Africa, Pretoria, Gauteng, South Africa

1. I think this study is important because it opens up opportunities to teach chemistry in context using IKS. Mixed methods were used. I think research question 1, What is the indigenous Chemistry knowledge possessed by pre-service science teachers? was addressed because the indigenous knowledge systems possessed by the students were identified.

**Thank you for pointing this out. We concur with  your comment.**

 2. The insight out of the second research question, How relevant is the indigenous Chemistry knowledge to Chemistry metacognition? was that Indigenous chemistry serves as prior knowledge in chemistry classrooms. I however thought that if the authors had selected an IKS conceptual framework to use in the study they were going to be able to unpack this insight more deeply. The insights may help us to clarify the understanding of IKS or indigenous chemistry in this case, that it is in fact applied chemistry and that the two types of knowledge are not different from each other

**Your observation on Indigenous Chemistry Knowledge being prior knowledge for Western Chemistry in the science classroom is on point. Thank you for your suggestion to use of IKS conceptual framework. It would have been intriguing to examine that aspect of using IKS conceptual framework. However, in this study, it appears slightly out of scope because it is the perfect theoretical framework to use on the influence of Indigenous Chemistry Knowledge on Chemistry metacognition. It is perfect as it focuses on the realization of the importance of context and culture in the learning of science. The indigenous chemistry knowledge is the learners’ culture and context which was  applied to chemistry metacognition (science education).**

 3. I also have an issue with branding some of the identified knowledge/practices as non-western. Let's take just one example of tree planting to avoid soil erosion. I think that is a universal agricultural practice in the west in Africa and everywhere. This is why I say it would have been good to have a conceptual framework that shows the the overlaps and differences in any of the two knowledge systems. The research question is however addressed.

**You have raised an important idea here. However, we are of the opinion that in the context of the study, this is indigenous knowledge as it is knowledge that is localised, practiced and was developed over centuries by a local people in a particular geographical area. The overlaps of western science knowledge and indigenous science knowledge is an indicator that science is one irrespective of geographic location only differing on culture and context. Hence the application of indigenous chemistry knowledge as prior knowledge for western chemistry knowledge.**

 4. The third research question, How effective is the indigenous Chemistry knowledge in Chemistry metacognition? pertained to the quantitative methods used. My question is how comparisons were made between data from focus group interviews (which I assume to be qualitative) and data from papers tests (whose scores can be used as quantitative data). Until the authors explain how they got quantitative data from focus group interviews to be able to calculate correlations, this part is difficult to accept as valid. There is an need to look at the methods again and provide the missing information. For example it is not sufficient to say data were analyzed through SPSS version 23 without specifying the particular comparison tool used.

**You have raised another perception here. However, the research was a mixed method research though it was mainly qualitative with quantitative to a smaller extent. There were two types of questionnaires that were used in the study. The first questionnaire was qualitative which was on Indigenous Chemistry Knowledge known by respondents. The second questionnaire was quantitative since it was a metacognitive awareness inventory which generates quantitative scores by design that were used before and after indigenous chemistry intervention. The metacognitive awareness was done using focus groups and paper and pen test for purposes of correlating the responses on metacognitive awareness as well as triangulation.  The purpose of the SPSS version 23 software was to convert the metacognitive awareness scores into bar graphs, line graphs and pie charts which were used to compare the scores before and after indigenous chemistry knowledge intervention**
